# Variation in Ventilator Allocation Guidelines by US State During the Coronavirus Disease 2019 Pandemic: A Systematic Review

**DOI:** 10.1001/jamanetworkopen.2020.12606

**Published:** 2020-06-19

**Authors:** Gina M. Piscitello, Esha M. Kapania, William D. Miller, Juan C. Rojas, Mark Siegler, William F. Parker

**Affiliations:** 1 Department of Medicine, Rush University, Chicago, Illinois; 2 Department of Pulmonary and Critical Care, University of Chicago, Chicago, Illinois; 3 Department of Medicine, University of Chicago, Chicago, Illinois; 4 MacLean Center for Clinical Medical Ethics, University of Chicago, Chicago, Illinois

## Abstract

**Importance:**

During the coronavirus disease 2019 pandemic, there may be too few ventilators to meet medical demands. It is unknown how many US states have ventilator allocation guidelines and how these state guidelines compare with one another.

**Objective:**

To evaluate the number of publicly available US state guidelines for ventilator allocation and the variation in state recommendations for how ventilator allocation decisions should occur and to assess whether unique criteria exist for pediatric patients.

**Evidence Review:**

This systematic review evaluated publicly available guidelines about ventilator allocation for all states in the US and in the District of Columbia using department of health websites for each state and internet searches. Documents with any discussion of a process to triage mechanical ventilatory support during a public health emergency were screened for inclusion. Articles were excluded if they did not include specific ventilator allocation recommendations, were in draft status, did not include their state department of health, or were not the most up-to-date guideline. All documents were individually assessed and reassessed by 2 independent reviewers from March 30 to April 2 and May 8 to 10, 2020.

**Findings:**

As of May 10, 2020, 26 states had publicly available ventilator guidelines, and 14 states had pediatric guidelines. Use of the Sequential Organ Failure Assessment score in the initial rank of adult patients was recommended in 15 state guidelines (58%), and assessment of limited life expectancy from underlying conditions or comorbidities was included in 6 state guidelines (23%). Priority was recommended for specific groups in the initial evaluation of patients in 6 states (23%) (ie, Illinois, Maryland, Massachusetts, Michigan, Pennsylvania, and Utah). Many states recommended exclusion criteria in adult (11 of 26 states [42%]) and pediatric (10 of 14 states [71%]) ventilator allocation. Withdrawal of mechanical ventilation from a patient to give to another if a shortage occurs was discussed in 22 of 26 adult guidelines (85%) and 9 of 14 pediatric guidelines (64%).

**Conclusions and Relevance:**

These findings suggest that although allocation guidelines for mechanical ventilatory support are essential in a public health emergency, only 26 US states provided public guidance on how this allocation should occur. Guidelines among states, including adjacent states, varied significantly and could cause inequity in the allocation of mechanical ventilatory support during a public health emergency, such as the coronavirus disease 2019 pandemic.

## Key Points

**Question:**

How many US states have ventilator allocation guidelines and how do these guidelines compare with one another?

**Findings:**

In this systematic review of publicly available US state guidelines about ventilator allocation, only 26 states provided guidance on how this allocation should occur, and their guidelines varied significantly.

**Meaning:**

These findings suggest significant variation in US state ventilator guidelines, which could cause inequity in allocation of mechanical ventilatory support during a public health emergency, such as the coronavirus disease 2019 pandemic.

## Teaser

This systematic review examines US state guidelines for ventilator allocation decision-making during the coronavirus disease 2019 pandemic.

## Introduction

Since the advent of worldwide mechanical ventilator use for patients with polio in the 1950s, ventilators have provided life-saving support to millions of people.^1^ In the US, ventilators have been widely available for the past 50 years. There have been concerns during the coronavirus disease 2019 (COVID-19) pandemic that the need for ventilators could exceed their availability, thus causing a widespread shortage of ventilators. In these circumstances, tragic choices would need to be made to determine who receives mechanical ventilatory support and who does not.^2^

Individual physicians, ethicists, medical societies, and US states have published multiple recommendations regarding how to allocate ventilators in a public health emergency and are largely in consensus that ventilators should be allocated to do the greatest good for the greatest number of people.^3,4,5,6,7,8,9,10,11^ However, it is currently unknown how many US states have translated these ethical standards into practical guidelines for how ventilator support should be allocated during a public health emergency. It is also unknown how the existing guidelines compare with one another regarding challenging questions, such as the method to rank patients in order of priority; whether it is acceptable to use age, chronic medical conditions, or estimates of remaining life-expectancy in priority scores; and whether it is ethical or legal to withdraw ventilatory therapy from one patient to provide it to another.^12^

This study was designed to evaluate the number of publicly available US state guidelines for ventilator allocation and to evaluate the variation in state recommendations for how ventilator allocation decisions should occur. We also aim to assess whether unique criteria exist for pediatric patients.

## Methods

### Study Design

Protocols for allocation of scarce resources, such as ventilators, should be available for public review and be transparent.^13,14,15^ Since these documents should be openly accessible, we searched for publicly available guidelines about ventilator allocation during a public health emergency for all 50 states in the US and the District of Columbia. Institutional review board review was not required, as the data did not involve human participants, as supported by the Common Rule. We completed a systematic review guided by the Preferred Reporting Items for Systematic Reviews and Meta-analyses (PRISMA) reporting guideline and searched for the most up-to-date guidelines through the internet using public health websites for each individual state and the Google internet search engine (Alphabet). Search terms included “ventilator,” “ventilator allocation,” “ventilator triage,” “scarce resource,” “crisis standard,” and “health emergency.” We did not complete a literature review, as documents found by this method may not be openly available to the public. Full details of the search strategy are explained in eAppendix 1 in the Supplement. We searched for adult and pediatric guidelines. Articles were excluded from review if they did not have specific ventilator allocation recommendations, were in draft status, did not include the state department of health, or were not the most recent allocation guideline available (eFigure 1 in the Supplement). All documents were individually assessed and reassessed by 2 independent reviewers (G.M.P. and E.M.K.) from March 30 to April 2 and May 8 to 10, 2020. Prior to reviewing protocols, each reviewer was trained to evaluate each screened article. Disagreement between reviewers occurred rarely, and each time it occurred, it was resolved by reevaluation of the protocols, which resulted in consensus agreement between reviewers.

### Data Collection

For each adult guideline, we collected data on scoring system; use of chronic conditions, age, or remaining life expectancy in the ranking of patients; exclusion criteria; identification of priority groups in the ranking of patients; use of tiebreakers; use of a triage committee; and discussion of the withdrawal of mechanical ventilation. How the protocol was created, whether the community was involved in its creation, whether state protocols were legally binding, whether validation of the guidelines had occurred, and whether the state planned to oversee implementation of the protocol were also evaluated. Data collected for pediatric guidelines included scoring system used, age of pediatric patients, exclusion criteria, discussion of withdrawal of mechanical ventilation, and whether pediatric patients were included with adults in allocation of ventilators. All reported data were current as of May 10, 2020.

## Results

We identified 44 guidelines, of which 17 (39%) met exclusion criteria (eFigure 1 in the Supplement). The remaining 27 guidelines (61%) met inclusion criteria as publicly available US state ventilator allocation protocols during a public health emergency with detailed recommendations (Table; eAppendix 2 in the Supplement). This included 26 state protocols and 1 pediatric-specific state protocol (eFigure 2 in the Supplement).

**Table.** Ventilator Allocation Guidelines

State	Exclusion criteria^a^	Rank tool		Adult	
		Adult	Pediatric	Initial tiebreakers	Discussed withdrawal
Alaska	No	SOFA and/or other parameters	No	Multicomponent strategy^b^	Yes
Arizona	No	SOFA^c^	No	No	Yes
California	Discussed, no recommendation made	SOFA	No	No	Yes
Colorado	Recommend against categorical exclusion criteria	Objective tool to measure severity of acute and chronic illness	Objective tool to measure severity of acute and chronic illness	Age <18 y, health care workers and first responders with a role in COVID-19 response	Yes (only after 14-21 d)
Connecticut	No	No	No	No	Yes
Illinois	No	No	No	No	Yes
Indiana	Yes	SOFA	SOFA	First-come, first-served	Yes
Iowa	No	SOFA	No	No	Yes
Kansas	Yes	SOFA	PELOD	Lottery or first-come, first-served	Yes
Louisiana	Yes	mSOFA	PELOD	No	No
Maryland	Yes	SOFA and life-limiting underlying conditions	PELOD 2 and comorbidities	Lottery or first come, first served	Yes
Massachusetts	Recommend against categorical exclusion criteria	SOFA and life-limiting underlying conditions	Prognosis for short-term survival and prognosis for long-term survival	Age	Yes
Michigan	Yes	SOFA	PELOD	Age, lottery, or first-come, first-served	Yes
Minnesota	No	SOFA and/or other parameters	No	Multicomponent strategy^b^	Yes
Nevada	No	SOFA, mSOFA, or qSOFA	No	No	No
New Mexico	No	SOFA^c^	No	No	No
New York	Yes	SOFA	Clinical judgment	Lottery	Yes
North Carolina	No	Severity assessment and survival likelihood	No	No	No
Oklahoma	No	SOFA and life-limiting underlying conditions	No	Age, essential personnel, people who put themselves in harm’s way	Yes
Oregon	Yes	mSOFA and clinical judgment	mSOFA or clinical judgment	Long-term prognosis	Yes
Pennsylvania	Recommend against categorical exclusion criteria	SOFA and life-limiting underlying conditions	PELOD and comorbidities	Age	Yes
South Carolina	Yes	SOFA	No	Comorbidities and age	Yes
Tennessee	Yes	SOFA or mSOFA	No	Multicomponent strategy^b^	Yes
Utah	Yes	Age, ASA score, and estimated survival	Clinical judgment	No	Yes
Vermont	No	SOFA	Clinician judgment	Multicomponent strategy^b^	Yes
Washington	Yes	mSOFA	PELOD 2	First-come, first-served	Yes

Abbreviations: ASA, American Society of Anesthesiologists Physical Status Classification System; COVID-19, coronavirus disease 2019; mSOFA, modified Sequential Organ Failure Assessment; PELOD, Pediatric Logistic Organ Dysfunction; qSOFA, quick Sequential Organ Failure Assessment; SOFA, Sequential Organ Failure Assessment.

^a^ Specific exclusion criteria differ by state.

^b^ Multicomponent tiebreaking strategy includes organ system function, duration of benefit or prognosis, duration of need, and response to mechanical ventilation, listed in relative order of importance.

^c^ Recommended to prioritize intensive care unit admission, but there was no specific score for ventilator allocation.

### Exclusion Criteria

**Figure 1.**
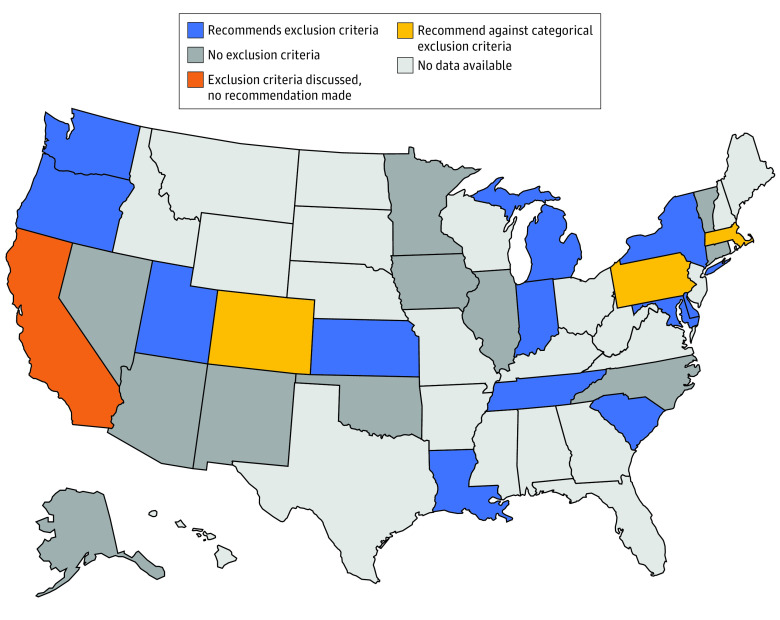
Exclusion Criteria for Adults

Of 26 state guidelines, 11 guidelines (42%) recommended exclusion criteria for ventilator allocation (Figure 1). Exclusion criteria are designed to remove patients from consideration if they have a very low chance of benefit from mechanical ventilation. Some state guidelines listed specific exclusion criteria, such as end-stage organ disease, irreversible severe neurologic injury or disease, metastatic malignant neoplasm, and severe dementia (eTable 1 in the Supplement). Additionally, 3 guidelines (12%) (ie, Colorado, Massachusetts, and Pennsylvania) specifically recommended against the use of categorical exclusion criteria.

### Scoring Systems

**Figure 2.**
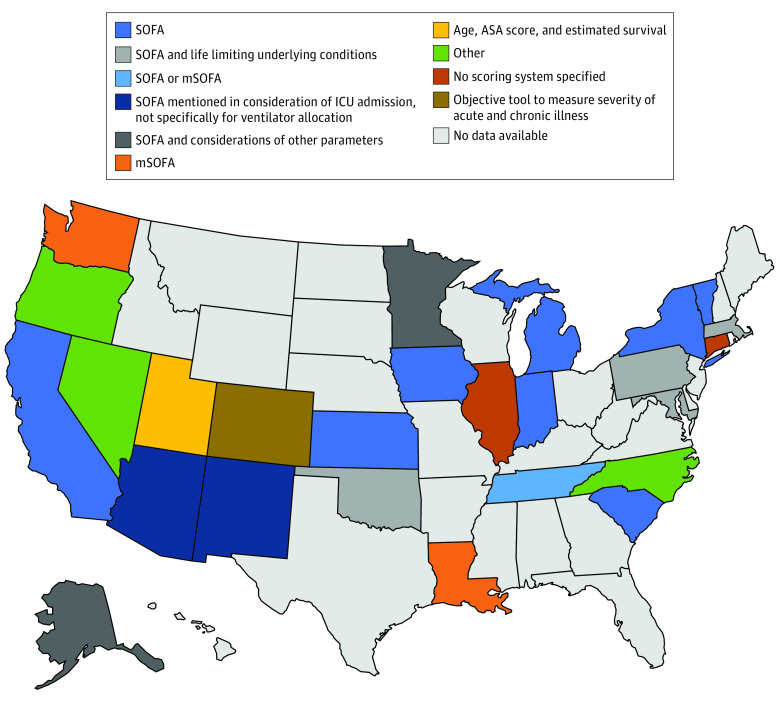
Ventilator Allocation Scoring Systems Recommended for Adult Patients Other category includes Oregon: modified Sequential Organ Failure Assessment (mSOFA) or clinical judgement mentioned in consideration of intensive care unit (ICU) admission, not specifically ventilator allocation; Nevada: SOFA, mSOFA, or quick SOFA (qSOFA); North Carolina: evaluation of severity of illness and likelihood of survival if provided the health care resources. ASA indicates American Society of Anesthesiologists Physical Status Classification System.

After the application of exclusion criteria, if present, 24 of 26 guidelines (92%) recommended an objective scoring system for the allocation of ventilators. The objective scoring systems used varied widely among states. The inclusion of the Sequential Organ Failure Assessment score was recommended in 15 guidelines (58%) for ventilator allocation. Other state guidelines recommended other scoring tools, including the modified Sequential Organ Failure Assessment (3 guidelines [12%]) and the American Society of Anesthesiologists Physical Status Classification System (1 guideline [4%]). Some state guidelines recommended using a multicomponent approach for ventilator allocation in addition to a calculated score, such as evaluation of age, estimated survival, and underlying comorbidities (1 guideline [4%]) and evaluation of decreased life expectancy related to underlying medical conditions or comorbidities (5 guidelines [19%]) (Figure 2).

### Priority for Specific Groups

**Figure 3.**
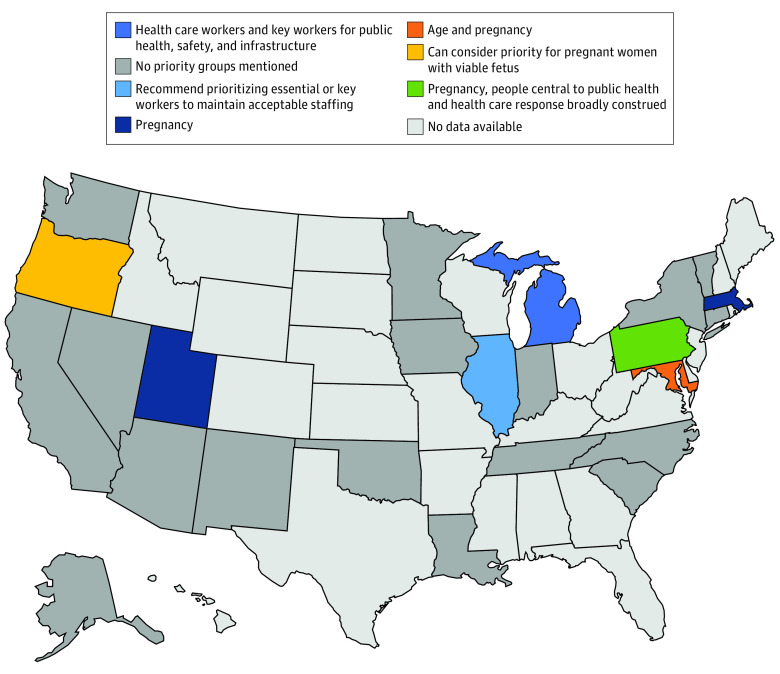
Priority Groups for Adults in the Initial Evaluation of Patients

Most state guidelines did not mention giving additional priority to specific groups of people. However, 6 guidelines (23%) (ie, Illinois, Maryland, Massachusetts, Michigan, Pennsylvania, and Utah) recommended giving priority advantages to particular groups in the initial scoring of patients. Maryland, Massachusetts, Pennsylvania, and Utah recommended giving priority to patients who are pregnant. In the Oregon guidelines, priority could be considered for patients who are pregnant. Additionally, 3 guidelines (12%) (ie, Illinois, Michigan, and Pennsylvania) recommended giving priority to health care workers and other key workers vital to the public health response. Younger age was recommended as a priority group in Maryland (Figure 3).

### Initial Tiebreakers

**Figure 4.**
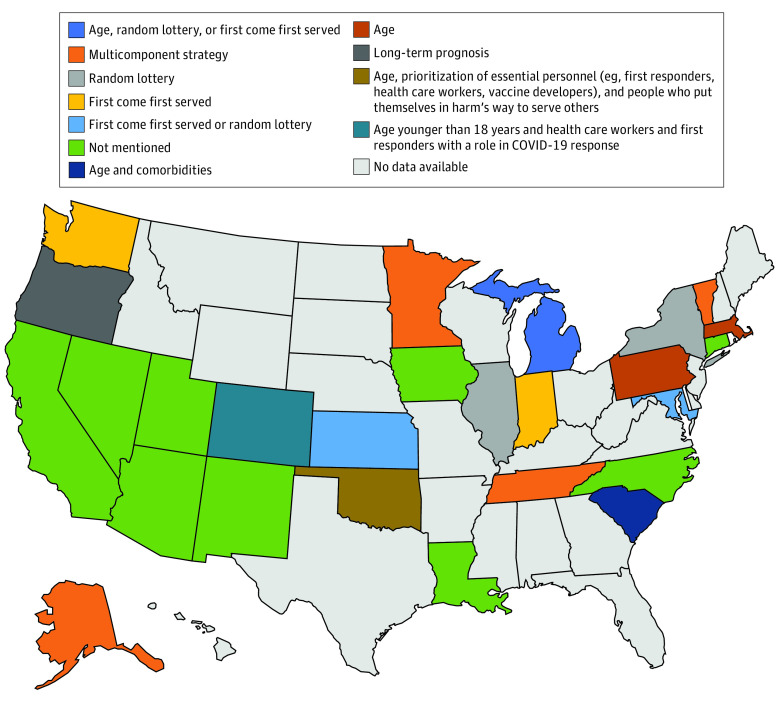
Initial Tiebreakers for Adults The multicomponent strategy in Alaska, Minnesota, Tennessee, and Vermont includes organ system function, duration of benefit or prognosis, duration of need, and response to mechanical ventilation, listed in relative order of importance.

If a tie exists after the initial scoring system is completed, 6 guidelines (23%) recommended including age as an initial tiebreaker to give priority to younger patients. Two guidelines (8%) recommended using status as a health care practitioner or other key worker vital to the public health response as a tiebreaker. Additionally, 5 guidelines (19%) recommended consideration of first-come, first-served as an initial tiebreaker (Figure 4).

### Withdrawal of Mechanical Ventilation

Withdrawal of mechanical ventilation from one patient to give to another may occur during a ventilator shortage when a patient with higher priority is in need of a ventilator. Withdrawal of mechanical ventilation was discussed in 22 guidelines (85%) (eFigure 3 in the Supplement).

### Decision-Making

Formation of a triage committee created by each hospital to implement protocols and make decisions regarding allocation of mechanical ventilators was recommended by 15 guidelines (58%). Triage committees were recommended by these states as bodies independent from patient care to promote objectivity, avoid conflicts in commitment to patients, and decrease moral distress experienced by clinicians providing direct patient care. They consist of multiple members, such as critical care physicians, nurses in leadership positions, and medical ethicists, and their recommended composition varied by state. The remaining 11 guidelines (42%) did not discuss who should make decisions about ventilator allocation (eFigure 4 in the Supplement).

### Pediatric Guidelines

Pediatric guidelines were found for only 14 states (eFigure 5 in the Supplement). The ages for what was considered *pediatric* varied by state (eTable 2 in the Supplement). Among these state guidelines, the most common scoring systems recommended for ventilator allocation included clinical judgment (2 guidelines [14%]) and the Pediatric Logistic Organ Dysfunction score (2 guidelines [14%]), while 2 guidelines (14%) recommended using evaluation of comorbidities in scoring systems (eTable 3 in the Supplement). Additionally, 7 guidelines (50%) discussed allowing children to be triaged with adults, and the age required to be triaged with adults varied by state (eTable 4 in the Supplement). Exclusion criteria were present in 10 guidelines (71%) (eTable 5 in the Supplement). Withdrawal of ventilatory support owing to scarce resources was discussed by 9 guidelines (64%), including 2 guidelines that required a minimum amount of time on a ventilator before reallocation could occur (eTable 6 in the Supplement).

### Creation and Composition of Guidelines

A total of 25 state guidelines (96%) reported that a committee was involved in creation of the protocol, with the composition of committees varying among states. Examples of committee members included physicians, nurses, medical ethicists, lawyers, and representatives from religious congregations. Additionally, 13 guidelines (50%) reported community involvement in the creation of the guideline, and 5 guidelines (19%) recommended community involvement but did not state whether it had yet occurred. No state guideline mentioned legislation to enforce use of the guideline. All guidelines offered guidance for hospitals to use its recommendations; however, no guideline explicitly discussed plans for state oversight to evaluate the use of the guideline in practice. No state guideline discussed whether validation of its ventilator allocation guideline was completed.

## Discussion

This systematic review found that 26 states had publicly available ventilator guidelines and 14 states had pediatric guidelines. There was significant variation in the states’ allocation guidelines regarding exclusion criteria, the predictive models used to determine priority scores, and the use of age, limited life expectancy, or chronic conditions to rank patients. Differences were observed in the use of scoring systems, with 6 distinct scoring mechanisms recommended for adults and 8 distinct scoring mechanisms recommended for children. Considerations of withdrawal of mechanical ventilation were discussed for most states with guidelines. Nearly half of states recommend the use of exclusion criteria. Priority groups were not often recommended. The use of initial tiebreakers for decision-making varied significantly among groups, with 8 distinct mechanisms recommended among 26 states.

The diversity of scoring systems revealed in this study is concerning, as it suggests access to mechanical ventilatory support would vary significantly by state in the event of a ventilator shortage. Not only did states use different predictive models for short-term survival, they made different choices regarding whether age, limited life expectancy, and chronic conditions were factored into their scoring system. As a result of these seemingly arbitrary choices, it is possible that in one state, a patient may receive mechanical ventilatory support, while in another state, the same patient would not. For instance, in Pennsylvania, a patient with a severe life-limiting condition with death likely within 1 year and a Sequential Organ Failure Assessment score of 6 would be considered intermediate priority for mechanical ventilatory support, while the same patient presenting to a hospital in neighboring New York would be designated high priority.^14,16^

Almost half of states with guidelines categorically excluded certain patients from ventilator support. Guidelines from 2014 support the notion that exclusion criteria can be ethically permissible in public health crises, as they are an objective and transparent determination.^5^ However, it is concerning that the discrete exclusion criteria used varied widely among states. In states with exclusion criteria, some patients would not be considered for a ventilator during a shortage even if their likelihood of acute survival was better than another patient who did not meet exclusion criteria. For example, in states that exclude patients with end-stage renal disease undergoing dialysis, a patient would be excluded from consideration even if they would otherwise be able to live on dialysis for years and may be eligible for a renal transplant that would extend their life span.^17,18,19^ Another concern about the use of exclusion criteria is that they have the potential to discriminate against certain populations, such as those with lower socioeconomic status who may have more comorbidities, people with disabilities, people with cognitive deficits (eg, dementia), or children with metabolic or chromosomal anomalies.^20,21^ Exclusion criteria also remove equitable access to mechanical ventilatory support, as some populations are automatically excluded from consideration without receiving an objective score to determine priority.^3^

Withdrawal of mechanical ventilation from one patient to provide ventilatory support to another patient with higher priority is a difficult decision that can be distressing for patients, families, staff, and the legal system.^3,12^ Withdrawal of ventilatory support can be ethically justified based on the sound principle of maximizing lives saved and the more unsettled principle that withholding and withdrawing ventilatory support are ethically equivalent for patients and clinicians.^5^ Although most states’ guidelines supported the notion of ventilator withdrawal during a public health crisis, they rarely provided clinical or ethical guidance on how these decisions should be discussed with families and alternate decision makers, how to support medical staff participating in the withdrawal, and whether decisions meet current states’ legal standards.^12,22^

We found that only 6 states recommended giving priority to selected groups in their initial scoring system, with 4 states giving priority to patients who are pregnant (ie, Maryland, Massachusetts, Pennsylvania, and Utah), 3 states giving priority to health care workers (ie, Illinois, Michigan, and Pennsylvania), and 1 state giving priority for younger patients (ie, Maryland).^16,23,24,25,26^ If a tie occurs after initial scoring is completed, 2 states (ie, Colorado and Oklahoma) recommended giving priority to health care workers and other essential personnel, and 6 states recommended giving priority to younger patients for the initial tiebreaker. Additionally, 5 states recommended consideration of a first-come, first-served system as an initial tiebreaker, which likely would prioritize patients with greater access to health care over those without. Giving priority to certain groups, such as health care practitioners, may help maintain a workforce of medical professionals who can continue to care for patients. It may also decrease the anxiety and distress of health care workers who are worried about their own risk of becoming sick.^14,27^ However, there are ethical concerns that essential worker groups, such as health care workers, should not be given priority over others. Some argue that health care workers who are sickened in a pandemic are unlikely to return to the workforce in time to assist in the immediate response, and there are significant ethical concerns about giving priority to groups with perceived higher social value. There is also debate about how broadly to define the pool of essential workers. The New York state guidelines classify essential workers as a broad category that can include hospital staff, firefighters, and police officers, and prioritizing this large of a group would diminish available ventilators for the rest of society. This would deny equitable access of mechanical ventilation throughout the population.^14^ While some guidelines called for age to be used in consideration in allocation decisions, age was not commonly implemented as a factor for initial triage decisions. Only 1 state considered it in their initial prioritization of patients.^23^

Although pediatric guidelines for ventilator allocation exist, they were found less often than adult criteria, and their allocation protocols and age criteria varied widely. One potential reason for the limited number of state pediatric scoring protocols may be related to the fact that pediatric scoring guidelines have not been validated for use in triage situations, so it is unknown what scoring system would be the most accurate.^14^ In addition, the uncertainty of how scoring protocols should vary according to the age of the child also likely contributes to fewer pediatric guidelines, as some states recommended adult scoring protocols for children to determine triage of ventilators. Regarding discussion of ventilator withdrawal, pediatric protocols discussed this less frequently than adult protocols, perhaps in part owing to the increased moral difficulty for medical staff to withdraw ventilators from children.

### Limitations

This study has some limitations, such as that only 26 states had publicly available guidelines to assess. It is possible that other states have guidelines they provide to individual hospitals in nonpublic ways. Nevertheless, we believe these numbers to be fairly accurate, as guidelines for allocation of scarce resources are recommended to be publicly available and transparent.^13,14,15^ Even with 48% of states not having public guidelines, our results are still illuminating, as more than 50% of states had guidelines that demonstrated significant variation in guidance provided. Another limitation is that this study evaluated only state guidelines and not specific hospital guidelines. It is unknown whether individual hospitals will choose to follow their state’s guidance in the event of a public health emergency, as individual state guidance may not be mandatory, and so these results may not accurately represent the allocation methods used by US hospitals in emergency situations. Additionally, this study is limited by the high likelihood that changes in state ventilator allocation strategies will continue to occur. Multiple states recently changed their protocols owing to concerns that they were discriminatory toward protected groups, such as people with disabilities or older age, after a bulletin released on March 28, 2020 from the Office for Civil Rights at the US Department of Health and Human Services,^21^ and other states may follow in changing their protocols.^28^ Lack of public input in some states during the creation of guidelines also may contribute to future changes as communities advocate for changes after becoming aware of the current guidelines.

## Conclusions

The findings of this systematic review suggest that although allocation guidelines for mechanical ventilatory support are essential in a public health emergency, only approximately half of US states currently provide public guidance about how this allocation should occur. Guidelines among states varied widely and could contribute to inequity in the allocation of mechanical ventilatory support throughout the US during a public health emergency, such as the COVID-19 pandemic.

## Notes

Supplement. **eAppendix 1.** Supplementary Methods **eAppendix 2.** State Ventilator Allocation Documents for Adults and Pediatric Patients Meeting Study Inclusion Criteria **eFigure 1.** Identification of US State Ventilator Allocation Protocols Meeting Inclusion Criteria **eFigure 2.** Ventilator Allocation Specific Guidance by US State **eFigure 3.** Withdrawal of Mechanical Ventilation Discussed for Adults **eFigure 4.** Triage Committee for Adults **eFigure 5.** Allocation Protocol for Pediatric Patients **eTable 1.** Specific Adult Exclusion Criteria **eTable 2.** Age of Pediatric Patients Per State Guidelines **eTable 3.** Pediatric Scoring Systems **eTable 4.** Pediatric Patients Triaged With Adults **eTable 5.** Exclusion Criteria for Patients Younger Than 18 Years **eTable 6.** Withdrawal from Mechanical Ventilation Discussed for Pediatric Patients
