# Supplementary material for: Variation in Ventilator Allocation Guidelines by US State During the Coronavirus Disease 2019 Pandemic: A Systematic Review
Source: JAMA Netw Open. 2020 Jun 19;3(6):e2012606. doi: 10.1001/jamanetworkopen.2020.12606 (PMC7305526; doi:10.1001/jamanetworkopen.2020.12606)
Supplement: Supplement. — eAppendix 1. Supplementary Methods eAppendix 2. State Ventilator Allocation Documents for Adults and Pediatric Patients Meeting Study Inclusion Criteria eFigure 1. Identification of US State Ventilator Allocation Protocols Meeting Inclusion Criteria eFigure 2. Ventilator Allocation Specific Guidance by US State eFigure 3. Withdrawal of Mechanical Ventilation Discussed for Adults eFigure 4. Triage Committee for Adults eFigure 5. Allocation Protocol for Pediatric Patients eTable 1. Specific Adult Exclusion Criteria eTable 2. Age of Pediatric Patients Per State Guidelines eTable 3. Pediatric Scoring Systems eTable 4. Pediatric Patients Triaged With Adults eTable 5. Exclusion Criteria for Patients Younger Than 18 Years eTable 6. Withdrawal from Mechanical Ventilation Discussed for Pediatric Patients [file jamanetwopen-3-e2012606-s001.pdf]

Supplementary Online Content

Piscitello GM, Kapania EM, Miller WD, Rojas JC, Siegler M, Parker WF. Variation in ventilator allocation guidelines by US state during the coronavirus disease 2019 pandemic: a systematic review. *JAMA Netw Open*. 2020;3(6):e2012606. doi:10.1001/jamanetworkopen.2020.12606

eAppendix 1. Supplementary Methods

eAppendix 2. State Ventilator Allocation Documents for Adults and Pediatric Patients Meeting Study Inclusion Criteria

eFigure 1. Identification of US State Ventilator Allocation Protocols Meeting Inclusion Criteria

eFigure 2. Ventilator Allocation Specific Guidance by US State

eFigure 3. Withdrawal of Mechanical Ventilation Discussed for Adults

eFigure 4. Triage Committee for Adults

eFigure 5. Allocation Protocol for Pediatric Patients

eTable 1. Specific Adult Exclusion Criteria

eTable 2. Age of Pediatric Patients Per State Guidelines

eTable 3. Pediatric Scoring Systems

eTable 4. Pediatric Patients Triaged With Adults

eTable 5. Exclusion Criteria for Patients Younger Than 18 Years

eTable 6. Withdrawal from Mechanical Ventilation Discussed for Pediatric Patients

This supplementary material has been provided by the authors to give readers additional information about their work.

© 2020 Piscitello GM et al. *JAMA Network Open.*

eAppendix 1. Supplementary Methods

Syntaxes Used in Internet Search:

Search Engine	Search Strategy
Google Search	ventilator OR ventilator allocation OR allocation OR ventilator triage OR triage OR scarce resource OR crisis standard OR health emergency
U.S. State Department of Health or Department of Public Health Website	ventilator OR ventilator allocation OR allocation OR ventilator triage OR triage OR scarce resource OR crisis standard OR health emergency

Search Strategy and Results for Google Search for the State of Colorado Ventilator Allocation Guidelines. No limits were used in search.

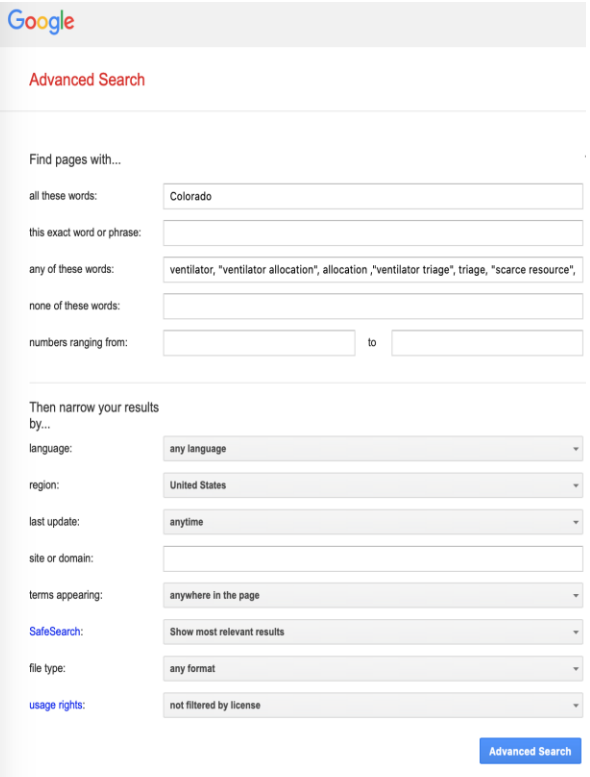

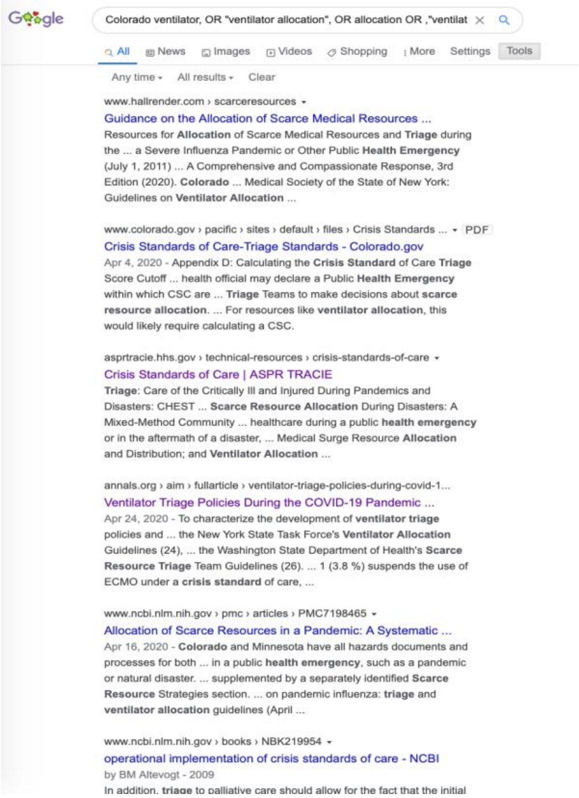

Dates of Search: All information sources were searched between March 30-April 2 and May 8-May 10, 2020.

No review protocol was created for evaluation of each state protocol. Our entire study design and search criteria are explained in our methods section on page 5-6 of the manuscript and this eMethods section.

Additional Studies: To be provided with a list of all screened protocols, please email Gina_Piscitello@rush.edu.

© 2020 Piscitello GM et al. *JAMA Network Open.*

eAppendix 2. State Ventilator Allocation Documents for Adults and Pediatric Patients Meeting Study Inclusion Criteria

Alaska

State of Alaska. Patient Care Strategies for Scarce Resource Situations [Internet]. 2020 [cited 2020 Apr 2];Available from: http://dhss.alaska.gov/dph/Epi/id/SiteAssets/Pages/HumanCoV/SOA_DHSS_CrisisStandardsOfCare.pdf

Arizona

Arizona Department of Health Services. Arizona Crisis Standards of Care Plan. A Comprehensive and Compassionate Response. 2nd Edition. [Internet]. 2018 [cited 2020 May 8];Available from: https://www.azdhs.gov/documents/preparedness/emergency-preparedness/response-plans/azcsc-plan.pdf

California

California Department of Public Health. Standards and Guidelines for Healthcare Surge During Emergencies - Foundational Knowledge [Internet]. [cited 2020 May 8];Available from: http://www.cidrap.umn.edu/sites/default/files/public/php/258/258_foundation.pdf

Colorado

Subject Matter Experts Advisor Panel for the Governors Expert Emergency Epidemic Response Committee. Crisis Standards of Care Guidelines for Hospitals for the COVID-19 Pandemic [Internet]. 2020 [cited 2020 May 10] ;Available from: https://www.colorado.gov/pacific/sites/default/files/Crisis%20Standards%20of%20Care%20Triage%20Standards-April%202020.pdf

Connecticut

Standards of Care Workgroup CT Department of Public Health. Standards of Care: Providing Health Care During A Prolonged Public Health Emergency [Internet]. 2010 [cited 2020 May 8];Available from: https://portal.ct.gov/-/media/Departments-and-Agencies/DPH/dph/legal/StandardsofCarefinalpdf.pdf?la=en

Illinois

State of Illinois - Illinois Department of Public Health. Guidelines on Emergency Preparedness for Hospitals During COVID-19 [Internet]. 2020 [cited 2020 May 8]. Available from: https://www.dph.illinois.gov/sites/default/files/Guidelines%20on%20Emergency%20Preparedness.pdf

© 2020 Piscitello GM et al. *JAMA Network Open.*

Indiana

Indiana State Department of Health Crisis Standards of Care Community Advisory Group. Crisis Standards of Patient Care Guidance with an Emphasis on Pandemic Influenza: Triage and Ventilator Allocation Guidelines [Internet]. 2014 [cited 2020 May 8];Available from: https://www.in.gov/isdh/files/Crisis_Standards_of_Care_signed_41814.pdf

Iowa

The Iowa Pandemic Influenza Ethics Committee. An Ethical Framework for Use in a Pandemic [Internet]. 2008 [cited 2020 May 8];Available from: http://publications.iowa.gov/17889/1/panflu_ehtical_guidelines_manual.pdf

Kansas

Kansas Department of Health and Environment. Toolkit for COVID-19 [Internet]. 2020 [cited 2020 May 8]; Available from: https://int.nyt.com/data/documenthelper/6847-kansas-triage-guidelines/02cb4c58460e57ea9f05/optimized/full.pdf#page=1

Louisiana

Louisiana Department of Health & Hospitals. ESF-8 Health & Medical Section State Hospital Crisis Standard of Care Guidelines in Disasters Version 14.0 [Internet]. 2018 [cited 2020 May 8];Available from: https://cdn.ymaws.com/www.lhaonline.org/resource/resmgr/imported/Louisiana%20CSOC%20Guidelines%20in%20Disasters.pdf

Maryland

Daugherty-Biddison L, Gwon H, Regenberg A, Schoch-Spana M, Toner E. Maryland Framework for the Allocation of Scarce Life-sustaining Medical Resources in a Catastrophic Public Health Emergency [Internet]. 2017 [cited 2020 May 8];Available from: https://www.law.umaryland.edu/media/SOL/pdfs/Programs/Health-Law/MHECN/ASR%20Framework_Final.pdf

Massachusetts

The Commonwealth of Massachusetts Department of Public Health. Crisis Standards of Care Planning Guidance for the COVID-19 Pandemic [Internet]. 2020 [cited 2020 May 8]. Available from: https://www.documentcloud.org/documents/6843353-Revised-Crisis-Standards-of-Care-Planning-Guidance.html

Michigan

State of Michigan Department of Community Health Office of Public Health Preparedness. Guidelines for Ethical Allocation of Scarce Medical Resources and Services During Public Health Emergencies in Michigan Version 2.0 [Internet]. 2012 [cited 2020 May 8];Available from: http://www.mimedicalethics.org/Documentation/Michigan%20DCH%20Ethical%20Scarce%20Resources%20Guidelines%20v2%20rev%20Nov%202012.0.pdf

Michigan Pediatric Protocol

Michigan Department of Health & Human Services. Planning for Children in Disasters: A Hospital Toolkit [Internet]. 2016 [cited 2020 May 8];Available from: https://www.michigan.gov/documents/mdch/Planning_for_Children_in_Disasters_15_495237_7.pdf

Minnesota

Minnesota Department of Health. Patient Care Strategies for Scare Resource Situations [Internet]. 2019 [cited 2020 May 8];Available from: https://www.health.state.mn.us/communities/ep/surge/crisis/standards.pdf

Nevada

Nevada Division of Public and Behavioral Health. Developing a Standard of Healthcare During Catastrophic Public Health Emergencies [Internet]. 2017 [cited 2020 May 8];Available from: https://files.asprtracie.hhs.gov/documents/nv-csc-plan-070317--final-508.pdf

New Mexico

New Mexico Department of Health. New Mexico Crisis Standards of Care [Internet]. 2018 [cited 2020 May 8];Available from: https://nmhealth.org/publication/view/plan/4877/

New York

New York State Task Force on Life and the Law and the Law New York State Department of Health. Ventilator Allocation Guidelines [Internet]. 2015 [cited 2020 May 8];Available from: https://www.health.ny.gov/regulations/task_force/reports_publications/docs/ventilator_guidelines.pdf

North Carolina

North Carolina Institute of Medicine in collaboration with the North Carolina Department of Health and Human Services, Division of Public Health. Stockpiling Solutions: North Carolina's Ethical Guidelines for an Influenza Pandemic [Internet]. 2007 [cited 2020 May

© 2020 Piscitello GM et al. *JAMA Network Open.*

8];Available from:
http://www.cidrap.umn.edu/sites/default/files/public/php/230/230_report.pdf

Oklahoma

Oklahoma State Department of Health. Hospital Crisis Standards of Care (Draft) [Internet]. Revised 4/7/2020 [cited 2020 May 8];Available from:
https://www.ok.gov/health2/documents/Hospital%20Crisis%20Standards%20of%20Care.pdf

Oregon

Oregon Health Authority et. al. Oregon Crisis Care Guidance [Internet]. 2018 [cited 2020 May 8];Available from: https://www.theoma.org/CrisisCare

Pennsylvania

Pennsylvania Department of Health. Interim Pennsylvania Standards of Care for Pandemic Guidelines Version 2 [Internet]. April 10, 2020 [cited 2020 May 8];Available from:
https://www.health.pa.gov/topics/Documents/Diseases%20and%20Conditions/COVID-19%20Interim%20Crisis%20Standards%20of%20Care.pdf

South Carolina

South Carolina Pandemic Influenza Ethics Task Force. South Carolina Prepares for Pandemic Influenza: An Ethical Perspective [Internet]. 2009 [cited 2020 May 8];Available from: https://www.scdhec.gov/sites/default/files/Library/CR-009538.pdf

Tennessee

Tennessee Altered Standards of Care Workgroup. Guidance for the Ethical Allocation of Scarce Resources during a Community-Wide Public Health Emergency as Declared by the Governor of Tennessee Version 1.6 [Internet]. 2016 [cited 2020 Mar 10];Available from: https://int.nyt.com/data/documenthelper/6851-tennessee-triage-guidelines/02cb4c58460e57ea9f05/optimized/full.pdf

Utah

Utah Department of Health and Utah Hospital Association. Utah Crisis Standards of Care Guidelines Version 2 [Internet]. 2018 [cited 2020 May 8];Available from:
https://health.utah.gov/wp-content/uploads/Final_Utah_Crisis_Standards_of_Care_011719-1.pdf

Vermont

© 2020 Piscitello GM et al. *JAMA Network Open.*

Vermont Department of Health. Vermont Crisis Standard of Care Plan Version 1.0 [Internet]. 2019 [cited 2020 Mar 30];Available from: https://www.healthvermont.gov/sites/default/files/documents/pdf/VT%20CSC%20Plan%2007-23-2019%20Final.pdf

Washington

Washington State Department of Health. Scarce Resource Management & Crisis Standards of Care [Internet]. 2020 [cited 2020 May 8];Available from: https://int.nyt.com/data/documenthelper/6853-washington-state-triage-guidel/02cb4c58460e57ea9f05/optimized/full.pdf#page=1

© 2020 Piscitello GM et al. *JAMA Network Open.*

eFigure 1. Identification of US State Ventilator Allocation Protocols Meeting Inclusion Criteria

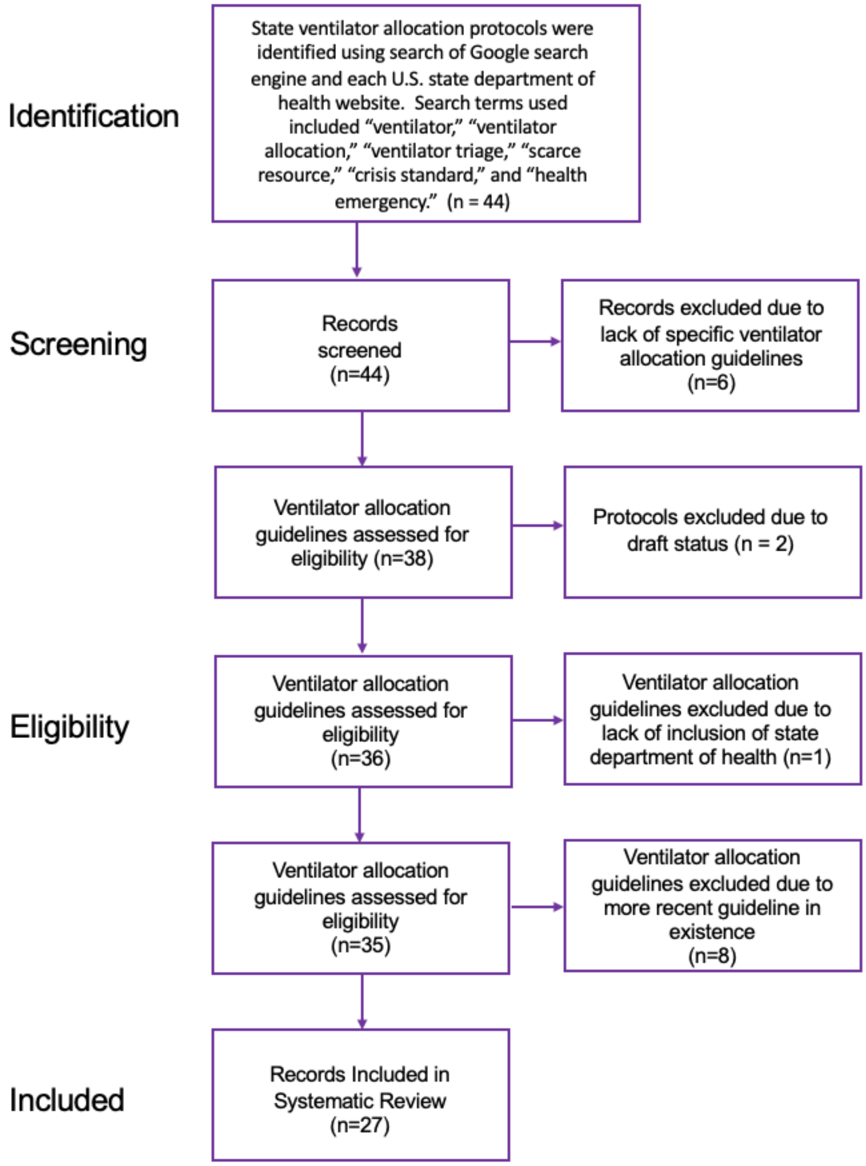

© 2020 Piscitello GM et al. *JAMA Network Open.*

eFigure 2. Ventilator Allocation Specific Guidance by US States

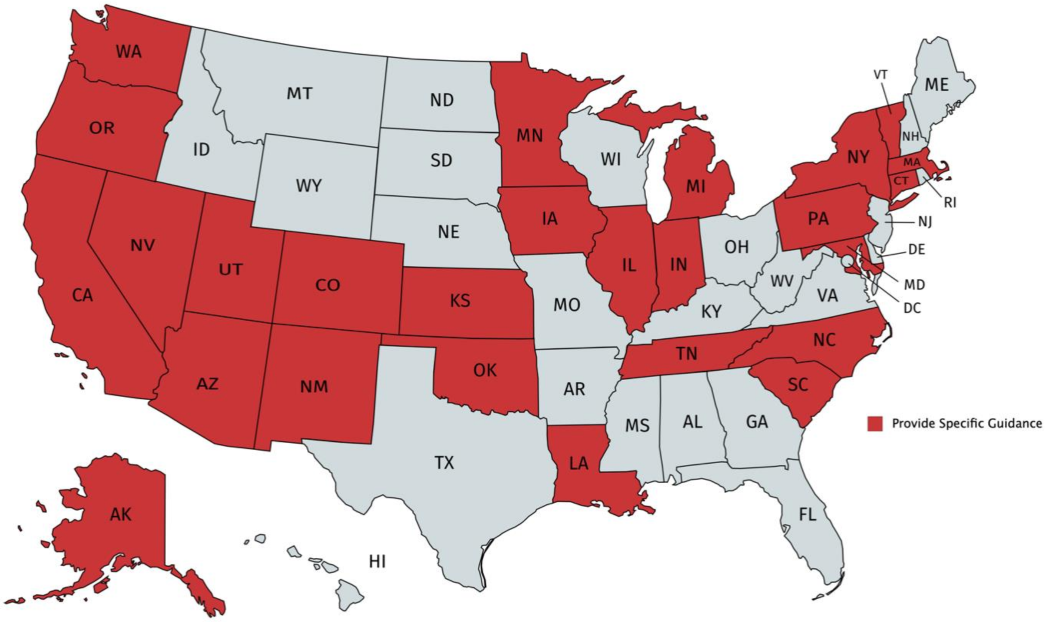

© 2020 Piscitello GM et al. *JAMA Network Open.*

eFigure 3. Withdrawal of Mechanical Ventilation Discussed for Adults

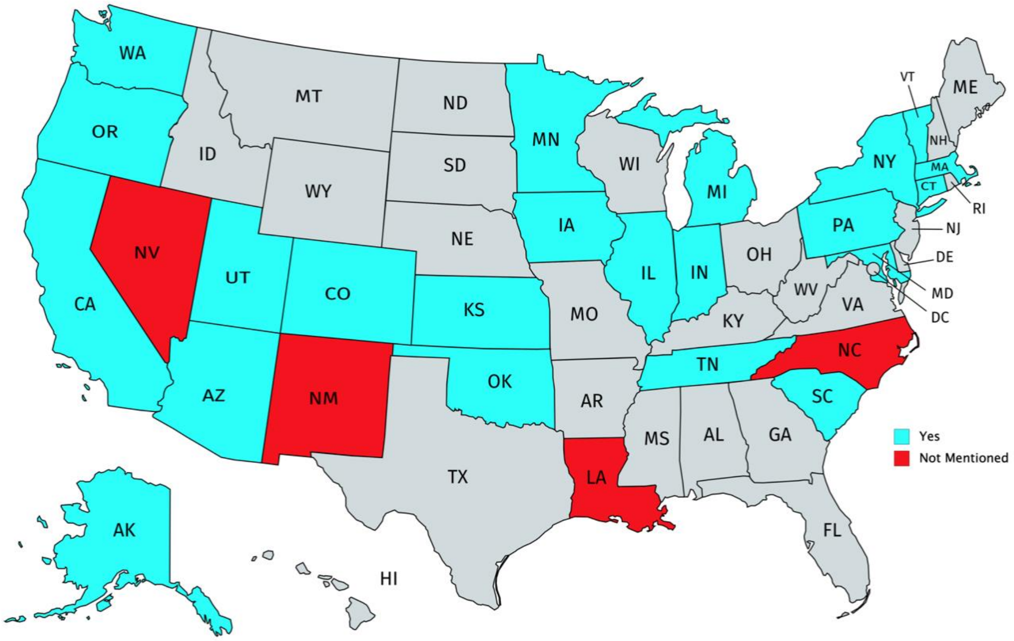

© 2020 Piscitello GM et al. *JAMA Network Open.*

eFigure 4. Triage Committee for Adults

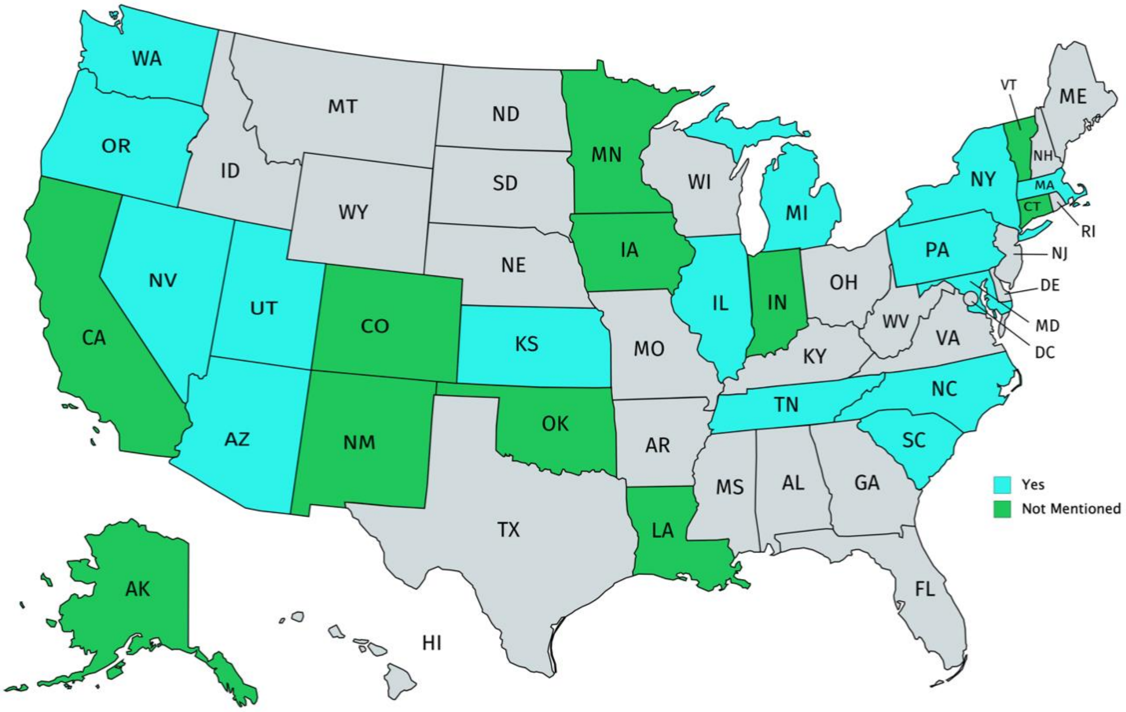

© 2020 Piscitello GM et al. *JAMA Network Open.*

eFigure 5. Allocation Protocol for Pediatric Patients

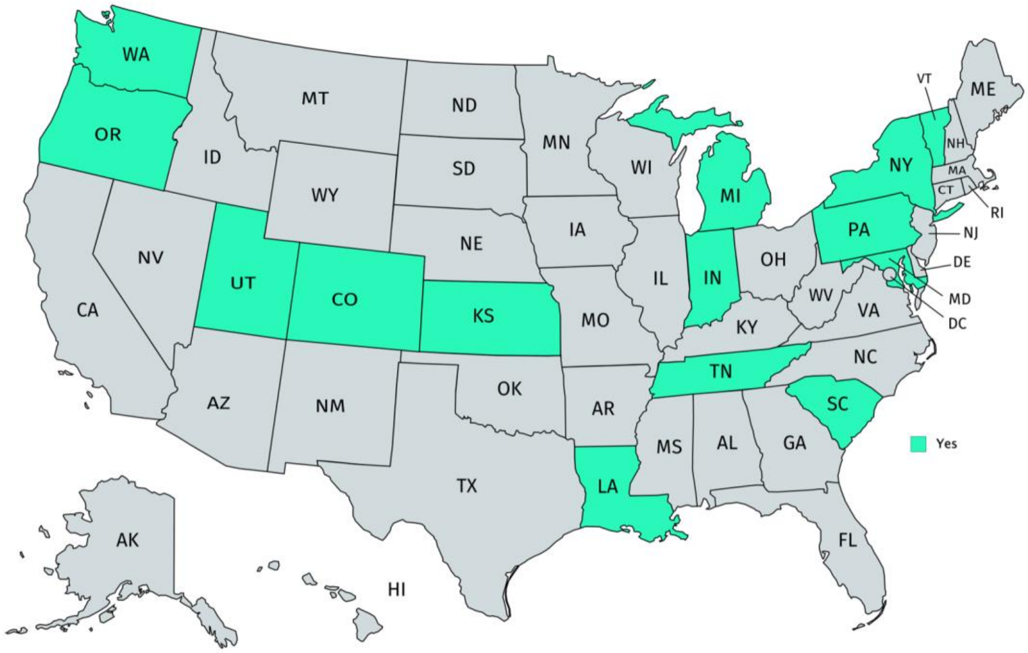

© 2020 Piscitello GM et al. *JAMA Network Open.*

eTable 1. Specific Adult Exclusion Criteria

Diagnosis	Number of States with Criteria
Severe burns	10
Cardiac arrest not responsive to advanced cardiac life support	9
End stage heart disease	9
End stage liver disease	9
End stage lung disease	8
Irreversible severe neurologic injury or disease	8
Metastatic malignancy	8
Severe trauma	5
Severe dementia	3
End stage renal disease (dialysis dependent)	4
Terminal condition with life expectancy < 6 Months	4
Advanced untreatable neuromuscular disease	4
Age >90	1

© 2020 Piscitello GM et al. *JAMA Network Open.*

eTable 2. Age of Pediatric Patients Per State Guidelines

Age	Number of States
Less than Age 18	7
Age 14 or Younger	2
Less than Age 14	1
Less than Age 12	1
2 months to Age 12	1
Age 2 to Age 12	1
Age Not Specified	1

© 2020 Piscitello GM et al. *JAMA Network Open.*

eTable 3. Pediatric Scoring Systems

Score	Number of States
Clinical Judgment	2
PELOD	2
mSOFA or Clinical Judgment	1
PELOD	1
PELOD 2	1
PELOD + Comorbidities	1
PELOD 2 + Comorbidities	1
SOFA	1
Score Not Specified	4

Pediatric Logistic Organ Dysfunction (PELOD), Modified Sequential Organ Failure Assessment (mSOFA), Sequential Organ Failure Assessment (SOFA)

© 2020 Piscitello GM et al. *JAMA Network Open.*

eTable 4. Pediatric Patients Triaged With Adults

Yes	Age of Pediatric Patients that Can be Triaged with Adults
Colorado	Less than Age 18
Indiana	2 Months to Age 12
Massachusetts	Less than Age 18
New York	Less than Age 18
Tennessee	14 and Older
Vermont	Age 2 and Older
Oregon	No Age Specified

© 2020 Piscitello GM et al. *JAMA Network Open.*

eTable 5. Exclusion Criteria for Patients Younger Than 18 Years

**Have Exclusion Criteria**
Indiana
Kansas
Maryland
Massachusetts
Michigan
New York
Oregon
Utah
Vermont
Washington

© 2020 Piscitello GM et al. *JAMA Network Open.*

eTable 6. Withdrawal from Mechanical Ventilation Discussed for Pediatric Patients

**Yes**	**Special Requirements**
Colorado	Can consider after 14-21 days on mechanical ventilatory support
Indiana	Can consider after 120 hours on mechanical ventilatory support
Kansas	-
Maryland	-
Massachusetts	-
Michigan	-
New York	-
Oregon	-
Washington	-

© 2020 Piscitello GM et al. *JAMA Network Open.*
